# Effects of therapeutic exercise on the motor function of adults with Down syndrome: a systematic review and meta-analysis

**DOI:** 10.1038/s41598-023-48179-1

**Published:** 2023-12-11

**Authors:** Melanie Méndez-Martínez, Eliana-Isabel Rodríguez-Grande

**Affiliations:** 1 Universidad del Rosario, Bogotá, Cundinamarca Colombia; 2 School of Medicine and Health Sciences, Universidad del Rosario, Carrera 24 N. 63D–69, Bogotá, Colombia

**Keywords:** Health care, Medical research

## Abstract

Therapeutic exercise exerts positive effects by mitigating or reducing the motor or cognitive changes that people with Down syndrome undergo throughout their life. There are no updated systematic reviews that integrate the evidence available in a way that facilitates decision-making for physical rehabilitation teams. This study therefore aimed to consolidate the information available and compare the effects of different types of physical exercise on the motor function of adults with DS. We conducted a systematic review and meta-analysis of randomized clinical trials and quasi-experimental studies. The literature search was performed between January 2023 and February 2023 using the PubMed, SCIELO, Epistemonikos, and Lilacs databases. Studies were selected according to pre-determined inclusion and exclusion criteria. The risk-of-bias assessment was performed using the risk-of-bias rating tool for randomized clinical trial (RoB) and the risk of bias of non-randomized comparative studies was assessed using the risk of bias in non-randomized studies of interventions (ROBINS-I) tool. Risk-of-bias assessment and meta-analyses were performed using the RevMan software package. Sixteen studies met the eligibility criteria for the qualitative synthesis and 4 were included in the meta-analyses. Combined exercise significantly increased muscle strength both in the upper limbs (SMD = 0.74 [95% CI 0.25–1.22]) and lower limbs (SMD = 0.56[95% CI 0.08–1.04]). Aerobic exercise improved spatiotemporal gait parameters. Aerobic exercise showed significant improvements in dynamic balance while combined exercise significantly increased dynamic and static balance. The certainty of the evidence was low to moderate for all outcomes. There was low and moderate certainty of evidence for the outcomes proposed in this review. However, therapeutic exercise could be effective in improving muscle strength and gait functionality.

## Introduction

Down syndrome (DS) is a medical condition caused by a genetic abnormality where chromosome 21 is either partially or completely duplicated^1^. It is the most common and prevalent genetic neurological disorder associated with intellectual disability and motor disorders characterized by hypotonia, ligamentous laxity, and limited muscle strength^2^, in addition to other cardiorespiratory, gastrointestinal, and immunological comorbidities^3^.

The motor changes in balance, strength, resistance, and mobility^2^ caused by DS have a direct effect on motor function, defined as the ability or capacity to learn, maintain, coordinate, and assume voluntary control of postures and movement patterns^4^. Therefore, DS affects quality of life and the ability to perform activities of daily living (ADL), thus increasing the dependency on other individuals and the adoption of a sedentary lifestyle in many cases^5^.

This is the reason why DS individuals require therapeutic interventions, especially rehabilitation, to improve their motor skills. Therapeutic exercise is included within these interventions, which the World Health Organization defines as “a variation of physical activity, aimed at reaching a pre-established goal, which is generally the improvement or maintenance of physical fitness or of the health condition”^6^. Exercise is characterized as being planned and repetitive which means that it is performed regularly^6^ and is divided into different types: aerobic exercise, where large muscle groups are exercised improving cardiovascular capacity; strength exercises; flexibility or stretching exercises; and neuromuscular exercises, including proprioception^7–10^, balance, and agility exercises^11^.

Scientific evidence of the effects of exercise in DS individuals is extensive in this regard. Multiple interventions have been identified in the literature that evaluate the effectiveness of exercise in water^1,2^, progressive resistance exercise^12,13^, continuous aerobic exercise^5,14–16^, specific modalities such as Nordic walking^17^, and combined exercise which is simultaneous aerobic and resistance training^18–20^ on different motor function outcomes, such as aerobic and functional capacity^2,5^, dynamic balance, muscular strength, endurance^1,12,14,18–20^, and gait^17^.

However, the many types of therapeutic exercises available in the literature as well as the many motor function outcomes in which the effectiveness of these types of exercise is measured impede rehabilitation teams’ decision-making when attempting to identify the type of exercise that, according to its prescription, is the most effective in improving these motor function outcomes.

Despite having a substantial amount of evidence on the effect of exercise in adult DS individuals, there is no accumulation of this evidence that accounts for the effect of different types of therapeutic exercise, mode of application, and in general, the prescription parameters of effective interventions. The integration of available evidence will facilitate decision-making for physical rehabilitation teams. This study therefore aimed to consolidate the information available and compare the effects of different types of physical exercise on the motor function of adult DS individuals.

## Methods

This review was conducted in accordance with Cochrane Handbook of Systematic Reviews of Interventions^21^ and the recommendations of the methodology proposed in the Preferred Reporting Items for Systematic Reviews and Meta-Analyses (PRISMA) guidelines^22^. This analysis was prospectively registered on Open Science Framework (OSF) and it is available in https://doi.org/10.17605/OSF.IO/MRKN2. Ethical and internal review board approval was not required because no human or animal subjects were involved.

### Study selection criteria

#### Participants

DS individuals aged 18 years or older. Studies that included the population in this review as well as populations with other characteristics, and studies whose results for DS individuals were not presented separately, were excluded.

#### Interventions

Application of any type of therapeutic exercise—either strength or resistance, aerobic, or neuromuscular exercise—with specific prescription parameters such as intensity, duration, and frequency.

#### Comparison

Control group with no exercise intervention, or another type of exercise intervention with another prescription parameters.

#### Outcome measures

Studies that did not include at least one of the outcomes proposed for this review were excluded. The outcomes prioritized in this review were as follows:

- **Primary:** Strength, defined as the ability of a muscle group to develop contraction against resistance^23^; balance or equilibrium, understood as the ability to maintain the body’s stability on each side of the axis^24^; and gait variables, defined as bipedal walking used to move from one place to another with minimal effort and energy consumption^25^.
- **Secondary:** Coordination, defined as the ability to execute and control movements^24^; posture, defined as the alignment of body segments during movement or a sustained situation^25^; and functional tasks, such as climbing stairs.

#### Designs

Experimental studies such as randomized or quasi-experimental clinical trials were included.

### Search methods for identification of studies

The literature search was carried out in the PubMed, Epistemonikos, SCIELO and Lilacs databases. The following algorithms were used to search for articles in English:

**Table:** 

DATABASE	ALGORITHM
PubMed	((((((((((((((Down syndrome) OR Trisomy 21)) AND Adult)) AND (((((((Exercise) OR Physiotherapy) OR Physical) OR Training) OR Neuromuscular exercise))) AND (((((((((Motor function) OR Functionality) OR Balance) OR Posture) OR Coordination) OR Gait) OR Strength)) NOT Physiology))
Epistemonikos	(((((((((basic physical abilities) OR (Speed)) OR (Resistance)) OR (Strength)) OR (Skill)) OR (Flexibility)) OR (Coordination)) OR (Balance)) OR (Agility)) AND ((((((((((therapeutic exercise) OR (physiotherapy)) OR (physical therapy)) OR (exercise)) OR (Endurance Training)) OR (Motion Therapy)) OR (Muscle Stretching Exercises)) OR (Plyometric Exercise)) OR (Resistance Training)) AND (((((Syndrome, Down) OR (Mongolism)) OR (Trisomy G)) OR (Down's Syndrome)) OR (Trisomy 21)))

The search was conducted between January 2023 to February 2023 and no filters by language or publication date were applied.

#### Other sources

Additionally, other sources of evidence were consulted to allow for the identification and analysis of published or unpublished literature (gray literature) that had not been detected through the systematic search. These other sources included manual searches in the reference list of the systematic reviews found through the search in the databases. Another search was performed through the L·OVE platform (Living Overview of Evidence) in the Down Syndrome section^26^ which let us review and compare the studies in the Epistemonikos database where an evidence matrix was built to, automatically, list the systematic reviews that share at least one study included as well as all the studies included in each of these reviews^27^.

#### Study selection

This was performed by two reviewers independently applying the selection criteria (MMM, YVC). Duplicate studies were initially merged into a bibliographic reference manager, followed by screening through the review of titles and abstracts to identify studies that included the population of interest for the present review, therapeutic exercise intervention, and at least one of the motor function outcomes. Subsequently, the full texts of the selected studies were retrieved, and after a comprehensive reading, studies were excluded based on their design, the population included, or because they did not include at least one of the motor function outcomes. A third reviewer (ERG) intervened to define whether or not studies for which there was no agreement should be included in this review.

#### Data extraction and management

This was conducted by two reviewers independently (MMM, YVC) in an Excel file. The following items were extracted: year of publication and authors; title; characteristics of participants such as age, sex, and number of participants per group; characteristics of the interventions applied, such as the type and mode of exercise, with their prescription of intensity, duration, and frequency; outcomes evaluated with their respective measuring instruments; and the results obtained by variable and group.

#### Risk-of-bias assessment

This was performed using the risk-of-bias (RoB) tool for randomized clinical trials^21^ based on seven domains, namely, sequence generation, allocation concealment, blinding of participants and personnel, blinding of outcome assessors, incomplete outcome data, selective outcome reporting, and “other aspects.” Each of these domains was assigned a rating of “low risk,” “high risk,” or “unclear risk.” The risk of bias of non-randomized comparative studies was assessed using the risk of bias in non-randomized studies of interventions (ROBINS-I) tool^5^.

The response options for each domain-level judgement are: (1) Low risk of bias; (2) Moderate risk of bias; (3) Serious risk of bias; (4) Critical risk of bias; and (5) No information on which to base a judgement about risk of bias for this domain. Finally, the option response for an overall risk of bias judgement using ROBINS-I are: (1) Low risk of bias (the study is comparable to a well-performed randomized trial); (2) Moderate risk of bias (the study provides sound evidence for a non-randomized study but cannot be considered comparable to a well-performed randomized trial); (3) Serious risk of bias (the study has some important problems); (4) Critical risk of bias (the study is too problematic to provide any useful evidence and should not be included in any synthesis); and (5) No information on which to base a judgement about risk of bias^5^. Risk of bias assessment figures were developed in RevMan 5.4^28^.

### Synthesis of data

The selected body of evidence was assessed according to the prioritized outcomes. Each outcome described the population’s features; parameters of the interventions, including the exercise mode applied, frequency, intensity, and duration of the interventions applied in the said studies; and the quantitative results achieved with their level of significance. The data were synthesized on a Microsoft Excel base, extracting data from the population’s features, randomization methods, outcome measures, duration of follow-up, and assessment methods from each study. The meta-analysis considered direct comparisons between the experimental group that performed the interventions (aerobic exercise and resistance exercise) and a control group that performed educational activities, recreational activities, or continuity with ADL or exercise interventions with different parameters or the comparison between different types of exercise.

Averages and standard deviations of the data available from the selected studies were extracted from the prioritized outcomes included in the studies. When the studies reported standard errors of the mean, the standard deviations were obtained by multiplying standard errors of the mean by the square root of the sample size. Standardized mean differences (SMD) and 95% confidence intervals (95% CI) were calculated to combine the results of the studies using different measures for the same concept or of studies presenting variability in its features.

Heterogeneity between trials was assessed using the chi-squared test, with a *p* value of < 0.05 considered statistically significant after due consideration of the value of I^2^ Heterogeneity was reported as low (I^2^ = 0%–50%) or high (I^2^ > 50%)^29,30^. The results were combined using the random effects model and the 95% CI was calculated. All data analysis were performed using the RevMan 5 software^28^.

#### Assessment of the certainty of evidence

This was performed using the Grading of Recommendations, Assessment, Development and Evaluations (GRADE) system^21^ for each outcome. This system specifies four levels of quality evidence: “High,” “Moderate,” “Low,” and “Very Low.” The level is determined by considering the risk of bias in the study, inconsistency, direction of evidence, precision of an effect estimate, and other considerations that include publication bias, whether or not the effect is large, the existence of confounding factors, and the dose–response gradient. These variables, except for “other considerations,” were evaluated on a three-level scale: “not serious,” “serious,” and “very serious.” For “other considerations,” the publication bias scale was classified as “not detected” or “strong suspicion”; large effect was graded as “no,” “large,” or “very large”; confounders were graded as “no,” “will reduce the demonstrated effect,” or “suggests a spurious effect”; and the dose–response gradient was classified as “yes” or “no.”.

## Results

### Search and selection of studies

**Figure 1:**
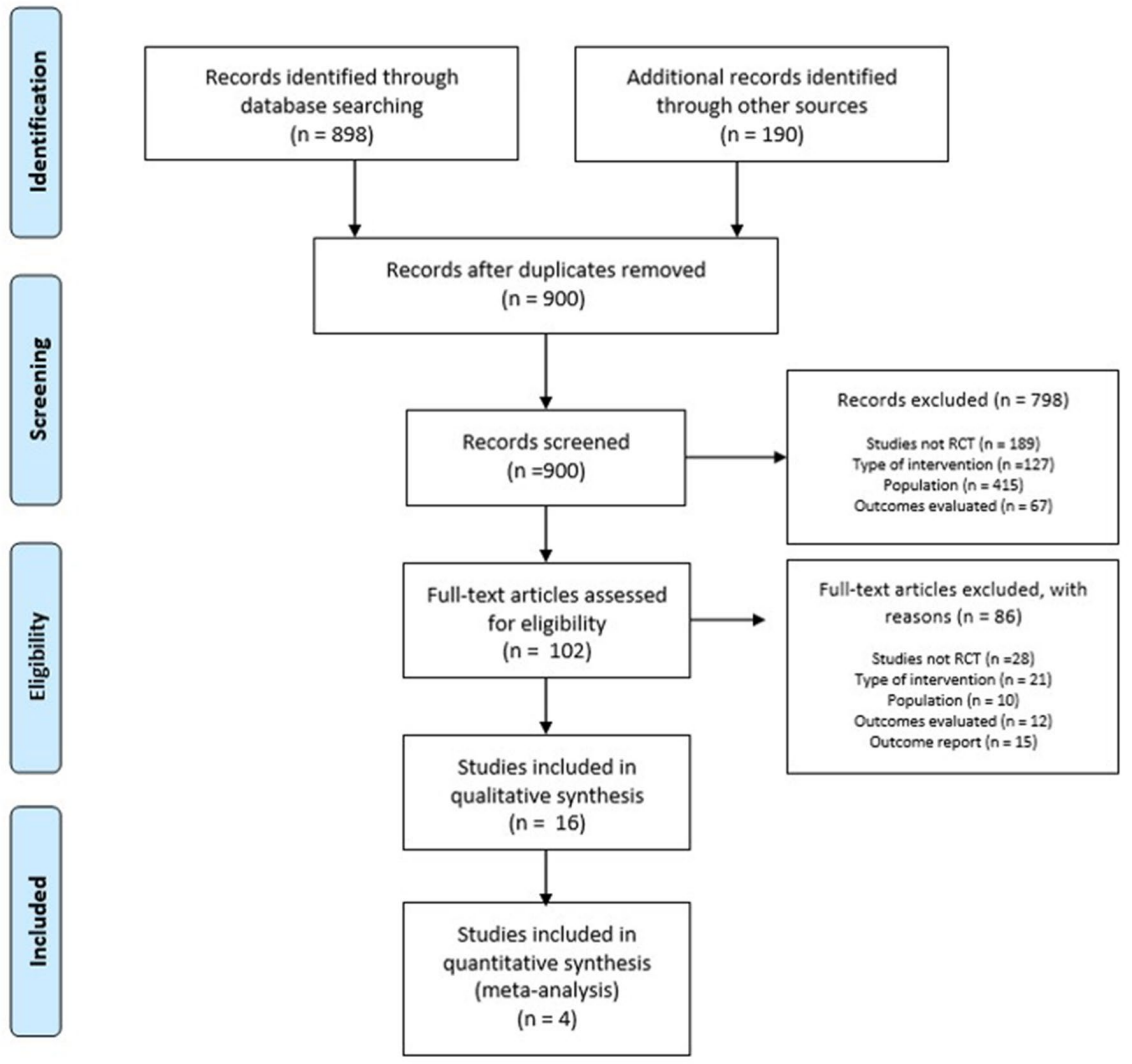
Flow diagram of the different phases of the investigation.

The electronic search yielded 898 studies, and 190 studies were obtained from other sources to yield a total of 1088. Of these, 188 studies were excluded owing to duplication and 798 were excluded after the review by titles and abstracts. In total, 102 studies were assessed in full text, of which 86 were excluded because they did not meet the eligibility criteria, mainly the study design, and because they did not include at least one of the outcomes prioritized in this review. Thus, only 16 studies met the eligibility criteria for the qualitative synthesis and 4 were included in the meta-analyses. This information is presented in a flowchart following the PRISMA model (Fig. 1).

### General characteristics of the studies

Table 1 shows that the studies selected for this review included a total of 402 participants aged 18–65 years. Of the 16 studies, 15 included the population aged 21–50 years^1,2,5,12,13,17,19,20,31–37^, with only 4^14,32,34,36^ including the population aged 60 years and older, and included the population aged 18–20^12,13,33,35–37^ years. The study with the smallest sample was the one by Post et al.^35^, with 11 participants, while that with the largest sample was the study by Rimmer et al.^20^, with 52 participants.

**Table 1:** General characteristic of the studies.

Study	Study design	Participants	Intervention	Dosage	Outcome measurement instrument	Results
Davis and Sinning (1987)^33^ United States of America	Quasi- experimental	**IG1:** 6 men with DS. Age: 20–38.2 years. IQ ranging 32–41 **IG2**: 6 men with mental disabilities without DS. Age 18.5–36.2 years. IQ ranging 33–57 **CG**: 6 undergraduate and postgraduate students. Age: 19–24.3 years. Above average IQ	**IG1 AND IG2:** Strength training under the supervision of graduates and graduates in physical education who were instructed in the procedures. Individual records of weight, series and repetitions were set **CG**: They exercised individually and recorded their own progress	**Mode:** bench press, triceps curls, and biceps curls with free weight **Intensity**: 6–8 repetitions. The amount of weight for each particular set was progressively increased as the subjects were able to exceed 8 repetitions **Duration**: 8 weeks **Frequency**: 3 times a week	**Elbow flexor strength:** Maximum voluntary contraction: supine, right arm in 90° elbow flexion, exert 2 maximum efforts against immovable resistance. In the same position, exert a force for 5 s against a series of loads ranging from 5 lb to approximately 90% of the maximum effort measured **Electromyography**: Electrodes are placed on the flexor muscle group at the elbow (biceps brachii). Integrated EMG and torque measurements of the elbow flexor muscle group were recorded simultaneously during maximal effort and step loading procedures	Only half of the subjects increased their maximum voluntary contraction as a result of the training, but there were no significant differences between groups P > 0.05 As expected, the post measurements of the group without disabilities experienced more improvement than the other two groups with disabilities, being statistically significant. *p* < 0.001
Carmeli et al. 2002^10^ Israel	Experimental	26 older adults aged 57–65 years Mild mental retardation IQ ranging 56–75 according to the Stanford Binet scale **IG**: 16 participants (10 women, 6 men) **CG**: 10 participants (6 women, 4 men)	**IG:** Aerobic exercise with treadmill walking Participants walked only between 9:30 and 11:30 am indoors under controlled conditions (23 °C, 40% humidity) **CG**: They were instructed not to change their daily activity level	**Mode:** Endless treadmill walk Intensity: Low resistance with 0% incline **Intensity**: Speed below the threshold for breathlessness but as fast as they could comfortably tolerate **Frequency**: 3 times/week **Duration**: 25 consecutive weeks. They initially walked for 10–15 min. The duration was gradually increased up to 45 min according to tolerance	**Dynamic balance and gait speed:** - Timed up and go **Muscle strength:** - Flexion (hamstrings) and extension (Quadriceps) of the knee in the isokinetic system (Biodex dynamometer) at speeds of 60°/s and 120°/s Data were collected for peak torque (ft/lb) (highest individual value of three peak efforts), peak torque percentage of body weight (ft/lb/kg), and average power (watts)	**Timed up and go: IG:** 25.9 ± 3 s. **CG:** 29.1 ± 3 s. Significant improvements between groups *p* < 0.05 **Muscle strength**: Significant differences are found in all three tests (maximum torque, % maximum torque of body weight and average power) of hamstrings and quadriceps in both men and women *p* < 0.01
Rimmer et al. (2004)^16^ United States of America	Experimental	52 adults with DS. Mean age 39.4 ± 6.4 **IG**: 30 participants **CG**: 22 participants. without intervention	**IG:** Cardio and strength exercises Exercise classes were supervised by a full-time registered clinical exercise physiologist and two assistants	**CARDIOVASCULAR TRAINING** **Mode:** Recumbent stepper, stationary cycle (recumbent and upright), treadmill and elliptical **Duration**: 15–20 min the first 2 weeks, 20–30 min the third and fourth weeks, 30 min from the fifth week onward for 12 weeks **Intensity**: 50%–70% of VO2 max. Monitored with cardiac monitors **STRENGTH TRAINING** **Mode**: seated bench and leg press, seated leg curl, triceps curl, seated shoulder press, seated row, push-up **Duration:** 15–20 min **Intensity:** initially 70% of 1-RM for a set of 10–20 reps. When participants were able to complete 20 reps for 2 consecutive sessions with proper lifting technique, the weight was increased by 10% of their 1-RM	**Strength:** 1-RM protocol according to the ACSM Leg press Chest press **Grip Strength:** Manual dynamometry	**Leg Press: IG:** 320 lb (87) 145.1 kg (39.4). **CG:** 208 lb (97) 94.3 kg (43.9). Significant differences *p* < 0.0001 **Chest Press: IG:** 100.7 lb (44.9) 45.6 kg (20.3). **CG:** 59.9 lb (33.6) 27.1 kg (15.2). Significant differences *p* < 0.0001 **Dynamometry:** **IG:** Right hand 22.0 (8.1); Left hand 21.6 (8.7) **CG:** Right hand 19.0 (7.7); Left hand 17.8 (7.0) Nonsignificant differences neither on the left nor on the right side *p* > 0.05
Tsimaras and Fotiadou (2004)^30^ Greece	Quasi-experimental	25 adults with DS all from the Center of Professional Learning of Thessaloniki **IG**: 15 participants. IQ range, 45–60. Age, 24.5 ± 3.9 years **CG**: 10 participants. (IQ range, 45– 59. Age 24.7 ± 2.7 years	**IG:** a 12-week Cardio and strength training	**Mode:** walking, slow running, and free gymnastic activities that mobilize large muscle groups (10 min) 15–20 min of training program activities: **1.** Two-foot ankle hop. **2.** Single-foot side-to side ankle hop. **3.** Tuck jump with knees up. **4.** Tuck jump with heel kick. **5.** Standing long jump. **6.** Standing jump over barrier. **7.** Single leg hops. **8.** Double leg hops. **9.** Standing on 1 foot. **10.** Rocking boats. **11.** Walking on line. **12.** Walking across 30-cm-wide balance beam. **13.** Walking across 20-cm-wide balance beam. **14.** Walking across 10-cm-wide balance beam Finally, 5-min recovery period **Frequency:** 3 times per week, **Duration:** 30–35 min/sesion	**Peak torque of knee extension and flexion:** Cybex II isokinetic dynamometer **Isokinetic muscle endurance of quadriceps muscles:** after 25 repeated maximum efforts at an angular velocity of 180° , and it was defined by the percentage of decline from the peak torque **Dynamic balance ability:** balance deck. Number of seconds the subject could remain standing on the platform of the stabilometer in durations of 30-, 45-, and 60-s intervals	**Isokinetic peak torque** of anterior (300, 180, 120° *p* < 0.001, 60°*p* < 0.01) and posterior (300, 180, 120° *p* < 0.01, 60° *p* < 0.01) femoral muscles significantly improved for the experiment group **The isokinetic endurance:** it showed a significant improvement (*p* < 0.01) after participation in the training program **Dynamic balance ability**: was significantly improved for the experiment group (30 s *p* < 0.01, 45 s and 60 s *p* < 0.001, No significant differences were found in any of the measurements for the control group For the initial and final measurements, no statistically significant differences were found between the 2 groups
Aguiar et al. (2008)^27^ Brazil	Quasi-experimental	**IG:** 21 men Age: 23.3 ± 2.1	Monitored aerobic exercise of adapted judo training for 16 weeks	**Mode:** Adapted Judo **Intensity**: Lactate threshold **Frequency**: 3 times/week **Duration**: 50 min/session	**Gross motor skills:** GMFM-88	The judo training program significantly (*P* < 0.05) improved the GMFM-88 index of young adults with DS
Shields et al. (2008)^8^ Australia	Experimental	20 adults. Age: 26.8 ± 7.8 years 13 men, 7 women **IG**: 9 participants **CG**: 11 participants 8 of the 20 participants worked at least 1 day/week in manual-type jobs (packing confectionery boxes, sorting and cutting clothes, and assembling car parts)	**IG:** Group progressive resistance training in a supervised community gym. The trainer kept a record for each participant of the number of repetitions and sets and the weight lifted/exercised in each session. Participants completed the program in a group, supervised by 2 accredited fitness trainers. Each trainer supervised the training of a subgroup of 2–3 participants **CG**: Continued with usual activities (work, free time, and leisure)	**Mode:** Progressive resistance training with machines: - Shoulder press - Seated chest press - Seated rowing - Seated leg press - Knee extension - Seated calf raise **Intensity**: Increased when 2 sets of 12 reps per exercise could be completed **Volume**: 2–3 sets of 10–12 reps per exercise to failure **Frequency**: 2 times a week **Duration**: 10 weeks **Density**: 2-min rest between sets	**Muscle performance:** - 1 RM: Chest press and leg press - Muscular resistance: repetitions of chest and leg press with 50% of 1RM **Physical function:** - Timed up and down stairs test - Grocery shelving task: Get up from a chair and take 2 bags of groceries to a bench located 2 m away. Each bag contains 10 items (410 g each, total weight of each bag 4.1 kg). Then they have to take the items out of the bag and stack them on a shelf at shoulder height	**1 RM Chest Press: IG:** 44.9 ± 15.2 kg. **CG:** 31.6 ± 13.3 kg **1 RM Leg Press: IG:** 96.2 ± 31.6 kg. **CG:** 82.2 ± 19.7 kg **Rep Chest Press: IG:** 25.9 ± 8.3. **CG** 17.5 ± 9.5 **Rep Leg Press: IG:** 46.8 ± 37.1. **CG:** 49.4 ± 27.6 **Timed up and down stairs: IG:** 14.4 ± 3.4 s. **CG:** 18.7 ± 6.5 s **Grocery shelving task: IG:** 67.5 ± 33.4 s. **CG:** pre 122.8 ± 84.0 s; post 110.7 ± 66.4 s Significant differences between groups in 1-RM chest press (*P* 0.08), chest press repetitions (*P* 0.002), and leg press repetitions (*P* 0.039) No significant differences between groups in the leg press 1RM test (*P* 0.90), timed up and go (*P* 0.12) or grocery shelving task test (*P* 0.11)
Cowley et al. 2011^9^ United States of America	Quasi- experimental	30 adults with mild intellectual disabilities. Age: 28 ± 8 years **IG**: 9 men and 10 women **CG**: 11 participants. 8 men and 3 women	**IG:** progressive resistance training Each participant worked one on one with a professional who supervised all the training sessions **CG**: Maintained normal daily activities	**Mode:** Leg extension, leg curl, leg press, shoulder press, chest press, bicep curl, and tricep curl exercises performed on exercise machines **Intensity**: 3 sets of 8–10 reps per exercise. The weight lifted by the subject was recorded during the training period and progressively increased to constantly overload the muscle **Frequency**: 2 days per week **Duration**: 10 weeks	**Isometric and isokinetic strength of knee extensors and flexors:** Biodex System 3 dynamometer - Maximum isometric peak torque: 3 series of 3 maximum contractions with knee extensors and flexors at a joint angle of 45°, 60°, and 75° with 3 min interval between series - Maximum isokinetic peak torque: 3 series of 5 maximum contractions with knee extensors and flexors at 60°/s with 3 min interval between series **Functional tasks of daily life:** - Time to get up from a chair at different heights (30, 38, or 43 cm) as quickly as possible to an upright position with trunk and legs straight, keeping arms crossed over the chest - Gait speed: Walk 7.62 m - Go up and down 10 steps as fast as possible without using the support handrail and alternating feet and then go down	**Isometric flexor strength:** Significant differences between groups *P* < 0.05 in the three degrees of movement (45°, 60°, and 75°) **Isometric strength extensors:** Significant differences in the IG in the three degrees of movement *P* < 0.05 **Flexors and extensors isokinetic strength Significant** differences in the IG *P* < 0.05 **Getting up from a chair at different heights**: No significant differences *P* > 0.05 in any of the chair heights (30 cm, 38 cm, 43 cm) or in the 5 repetitions **10-step ascent and descent**: Significant differences in the IG *P* < 0.05 in both tests. **IG** Ascent: .83 SD 1.19. **CG**: 5.10 SD 1.19. GI descent: 4.38 SD 1.19. CG: 6.23 SD 2.80 **Gait speed**: **IG**: 1.72 SD 0.20. **CG**: 1.71 SD 0.24. No significant differences *P* > 0.05
Mendonca et al. (2011)^15^ Portugal	Quasi- experimental	**IG1:** 13 participants (10 men, 3 women) with DS. Age: 36.5 ± 5.5 years **IG2**: 12 participants (9 men, 3 women) without disabilities. Age: 38.7 ± 8.3 years	Combined resistance and strength exercise training The exercise sessions were supervised by an exercise physiologist and an assistant	**ENDURANCE TRAINING** **Mode:** Walk or run on a treadmill **Intensity**: target heart rate compatible with 65% (first three weeks) at 85% of VO2peak. Monitored with fc/participant clock **Duration**: 30 min. 12 weeks **Frequency**: 3 days/week **STRENGTH TRAINING** **Mode:** repeating a set of 9 exercises twice with < 30 s of rest between them. Train with leg press, chest and shoulder press, vertical pull, lower back, leg extension, bicep curl, and tricep curl. In addition, 1 set of 15 repetitions of abdominal push-ups in each rotation **Intensity**: 10% increase in 12-RM load when participants were able to complete 14 reps for 2 consecutive sessions with proper technique **Frequency**: 2 days/week	**Muscle strength:** 12-RM protocol on variable resistance machines - Leg press - Chest press - Vertical traction - Lower back - Leg extension Each participant was asked to perform 15 reps with relatively light resistance followed by 30 s of recovery. Resistance was then increased, and each participant performed a maximum of 5 sets of 12 repetitions until the 12-RM was reached. The recovery period between sets was exactly 2 min, and increments of 2.5–5 kg were used as each participant approached fatigue. The 12-RM was defined as the maximum load lifted through a full range of motion for a total of 12 repetitions. For most participants, the 12-RM was determined in 3–4 attempts	**Leg Press: IG1:** 110.2 ± 52.6. **IG2:** 171.3 ± 56.5 **Chest Press: IG1:** 35.3 ± 12.2. **IG2:** 51.3 ± 21.0 **Vertical Traction: IG1:** 39.2 ± 14.1. **IG2:** 59.4 ± 15.3 **Lower Back: IG1:** 35.6 ± 7.4. **IG2:** 51.9 ± 19.3 **Leg Extension: IG1:** 30.1 ± 10.3. **IG2:** 52.7 ± 17.6 Participants with Down syndrome showed lower muscle strength than participants without disabilities in all dynamic exercises, both before and after training Training was highly efficient in obtaining generalized improvements for 12-RM in both groups (*P* < 0.05). The magnitude of these improvements was similar between participants with and without Down syndrome
Boer and Moss, (2016)^5^ South Africa	Experimental	42 adults. Age 33.8 ± 8.6 (25 men, 17 women) **IG1**: 13 participants **IG2**: 13 participants **CG**: 16 participants	**IG1:** Continuous aerobic training (CAT) on a bicycle or treadmill **IG2**: Interval training (IT) with 10–30-s sprints on a bike or treadmill The two **IGs** performed the intervention under the supervision of a licensed sport scientist and exercise physiologist in a 2:6 (professional:participants) ratio **CG**: No intervention	**Duration:** 12 weeks. 30-min sessions the first six weeks (5-min warm-up, 20-min central act, 5-min cool down), the last six weeks’ sessions were increased by 5 min for the central activity Intensity: Warm up and cool down at 4 km/h Frequency: 3 times/week **IG1: CAT** **- Mode:** Continuous aerobic training by bicycle (50%) or treadmill (50%) **- Central act intensity:** first six weeks 70%–80% VO2peak, last 6 weeks 85% VO2peak **IG2: IT** - **Mode**: Interval aerobic training on a bicycle (50%) or treadmill (50%) - **Intensity**: 10–30-s max sprints with 90-s low cadence, low intensity gait, or bike	**Grip strength:** - Manual dynamometry in the dominant hand **Lower Body Strength:** - Sit-to-stand test **Agility and dynamic balance:** - 8-ft up and go **Aerobic capacity and functional ability:** - 6MWD	**Grip strength:** **CAT:** 26.1 kg (7.9). **IT:** 29.9 kg (8.9). **CG:** 25.5 kg (9.1). No significant differences between groups *P* = 0.57 **Lower Body Strength:** **CAT**: 15.2 (1.8). IT: 15.5 (1.8). CG: 13.3 (2.3). Significant improvements between groups *P* = 0.01 and only in the CAT group compared to the control group (*P* < 0.05) **Agility and balance:** **CAT**: 4.8 s (0.9). IT: 4.9 s (1.1). CG: 6.2 s (1.3). Significant improvements between groups *P* = 0.03 and only in the CAT group compared to the control group (*P* < 0.05) **Aerobic capacity and functional ability:** **CAT**: 563.2 m (74.9). **IT**: 562.6 m (81.7). **CG**: 495.9 m (85.2). Significant improvements between groups *P* = 0.01 and only in the CAT group compared to the control group (*P* < 0.05)
Silva et al. (2017)^32^ Portugal	Experimental	27 adults aged 18–60 years **IG**: 14 participants **CG**: 13 participants	**IG:** Wii-based exercise program that included training games for aerobic endurance, balance, and isometric strength **CG**: They completed their usual daily activities (usual treatment) at their occupational center, such as rehabilitation, life skills training, and art-related activities	**Mode:** Aerobic exercise through a Wii-based exercise program. Individual sessions or with another participant (half of the sessions in each format) **Frequency**: 3 sessions per week **Duration**: 2 months	**Physical aptitude:** Eurofit test battery: - Limb movement speed (Plate Tapping Test) - Static arm strength (Handgrip Test) - Running speed and agility (Shuttle Run) - Balance (Flamingo Balance) - Flexibility (Sit and Reach) - Explosive power of the legs (Standing Broad) - Trunk Strength (30-s Sit-ups) - Muscular resistance (Bent Arm Hang) **Functional mobility:** - Timed Up and Go - Response speed subtest of the Bruininks–Oseretsky Motor Competence Test First Edition **Motor skills**: - Beanbag Overhead	Significant improvements in the GI in the Handgrip test (**IG:** 25.42 (5.53). **CG:** 23.92 (6.45) *P* 0,025), in the sit and reach (**IG:** 36.92 (7.22). **CG:** 29.46 (10.53) *P* 0,014), in the standing broad (**IG:** 99.33 (29.49). **CG:** 90.69 (35.20) *P* < 0,001) and in the Bruininks–Oseretsky First Edition test (**IG**: 4.67 (2.81). **CG**: 4.77 (2.17) *P* 0.028) Significant differences between groups were identified in the plate tapping test (*P* 0.045), shuttle run (*P* 0.014), sit and reach (*P* 0.027), standing broad (*P* 0.003), 30-s sit-ups (*P* 0.040) and timed up and go (*P* 0.049) No significant differences in the handgrip test (*P* 0.837), flamingo balance (*P* 0.477), bent arm hang (*P* 0.086), Bruininks–Oseretsky First Edition (*P* 0.265), neither in the beanbag overhead nor in the hand right P 0.150 nor in the left *P* 0.083
Boer and de Beer (2019)^2^ South Africa	Quasi- experimental	23 adults. Age 31.4 ± 7.4 years **IG:** 13 participants (8 men, 5 women) **CG:** 10 participants (5 men, 5 women)	**IG:** Aerobic exercise in aquatic environment. Aquatic training. Sessions controlled and monitored by test instructors and senior Human Movement Sciences students (approximately one test instructor per two participants) **CG**: No intervention additional to ADLs	**Mode:** Aquatic training with arm circle exercises, lateral twists, walk in place, run in place, water scoops, lateral leg raises, back flutter kick, stomach flutter kick, jumping jacks, knee twists, side shift, squat jumps, lunge jumps, and longer jog, 1.4-m-deep pool **Duration**: 6 weeks, 35 min the first 3 weeks, 45 min the last 3 weeks. Consider a 3-min warm up and 2-min cool down **Frequency**: 3 times/week	**Static Balance:** - Balance on one leg **Dynamic Balance:** - Walk on a balance beam **Functional fitness:** - 6MWD - 8-ft up and go **Muscular strength:** - Sit-to-stand test -Curl‐up modified -Isometric push-up	**Static Balance:** No significant differences *P* > 0.05 in the static balance **IG**: 6.6 (3.5). **CG**: 5.1 (3.6), nor dynamic **IG**: 5.6 (0.8). **CG**: 4.6 (2.1) **Functional fitness**: Significant differences between groups *P* < 0.05 for the 6MWD test: **IG**: 602.1(98.7). **CG**: 519. 9 (111.9). No significant differences *P* > 0.05 in the 8-ft up and go test: **IG**: .3 (0.9). **CG**: .5 (0.9) **Muscle strength:** Significant differences between groups *P* < 0.05 for sit-to-stand test GI: 14.5 (2.2). CG: 13.0 (1.8) and modified curl up GI: 37.9 (30.1). CG: 20.0 (28.3). Nonsignificant differences *P* > 0.05 for isometric push-up **IG:** 82.2 (50.9). **CG:** 36.5 (32.5)
Skiba et al. (2019)^13^ Polonia	Experimental	22 adults aged 25– 40 years, with moderate intellectual disability (IQ: 36–51). 11 men, 11 women **IG**: 11 participants. **CG:** 11 participants	**IG:** Aerobic exercise with Nordic walking training program. The exercises were performed by a physiotherapist, who was a qualified Nordic walking instructor **CG**: Did not undergo any training	**Mode:** Brisk Nordic walking **Intensity**: Progressed over the course of the training sessions **Frequency**: 3 times a week **Duration:** 45 min	**Spatiotemporal parameters (step and stride length and speed) and maximum values of angles in the ankle, knee, hip, and shoulder joints in different phases of gait:** Using the Vicon 250 Optoelectronic System for Three-Dimensional Motion Analysis	**Gait parameters:** Significant differences in the right (*P* 0.002) and left (*P* 0.038) step length as well as for the right (*P* 0.002) and left (*P* 0.001) stride length. Regarding speed, only significant changes in right leg (*P* 0.011) **Angular values:** Significant changes for the right ankle (*P* 0.044) in support phase. Significant changes in the left knee, with increased flexion in the phase of medium support (*P* 0.002), terminal support (*P* 0.017) and initial sway (*P* 0.004). The hip does not present significant changes in the right or left leg during the initial contact (*P* 0.649–0.755), pre-swing (*P* 0.054–0.165) or terminal sway (*P* 0.738–0.896) Significant differences in the movement of the pelvis in the sagittal plane in the medium support phase for the right limb (*P* 0.038) and in the initial sway phase for the left limb (*P* 0.043). In the frontal plane, there were significant differences in the movement of the pelvis at the maximum point of movement of the left limb (*P* 0.027) and at the minimum point of movement of the right (*P* 0.002). In the transverse plane there are no significant differences in the right or left leg
Boer (2020)^1^ South Africa	Experimental	26 adults. Age 32.7 ± 6 years (13 men, 13 women) **IG:** 13 participants **CG:** 13 participants	**IG:** Aerobic exercise in aquatic environment. Freestyle swimming training, accompanied by lively music and strictly controlled by the main test instructor using a whistle **CG**: No structured intervention	**Mode:** Freestyle swimming in a 12-m long and 1.4-m deep pool. Swim a certain length of the pool and rest while a partner completes another length in the same lane. As soon as the partner reaches the middle of the lane, the other participant is instructed to swim **Frequency:** 3 times/week **Duration:** 20 min the first 4 weeks, 26 min the last 4 weeks	**Static Balance:** - Balance on one leg **Dynamic Balance**: - Walk on a balance beam **Functional fitness**: - 6MWD - 8-ft up and go **Muscular strength**: - Sit-to-stand test -Curl‐up modified -Isometric push‐up	**Static Balance:** No significant differences (*P* > 0.05) in **IG** static balance: 5.9 (3.3). CG: 5.5 (4.1). Significant differences (*P* < 0.05) between groups in **IG** dynamic balance: 5.3 (1.2). CG: 3.5 (2.6) **Functional fitness:** No significant differences (*P* > 0.05) for the 6MWD **IG** test: 553.8 (106.9). **CG**: 503.1 (118.7). Significant differences between groups (*P* < 0.05) in the 8-ft up and go test: **IG**: 5.4 (1.0). **CG**: 6.0 (0.9) **Muscle strength:** Significant differences between groups for the three tests *P* < 0.05: Sit-to-stand test: **IG**: 14.3 (1.6). **CG**: 13.6 (1.6). Modified curl up: **IG:** 33.3 (30.1). **CG:** 16.6 (22.1). Isometric push up: **IG:** 79.8 (41.9). **CG:** 47.3 (35.1)
Perrot et al. (2021)^28^ France	Experimental	12 participants. Age: 35 to 64, **IG:** 6 participants, mean age: 49.3 **CG:** 6 participants, mean age: 51.4	**IG:** Wii based exercise program using the Nintendo Wii, a video game console with integrated motion-sensitive technology and the Nintendo Wii Balance Board to record weight and center of pressure trajectory. Each training session was supervised by the master student in adapted physical activity **CG:** no lifestyle changes	**Mode:** Wii-based exercise program First period: Wii sports (Wii Tennis and Wii Bowling) Second period: Wii Fit Plus using the balance board to play Wii Soccer Headers, Wii Ski Jump, Wii Hula Hoop, and the Wii Marbles games **Duration:** 1 h/session for 14 weeks **Frequency:** 2 sessions/week	**Functional mobility:** - TUG - TUDS **Muscular endurance:** The 30-s chair stand test **Physical fitness:** 6-Minute Walk Test	Improvement in the IG was observed with large effect sizes for functional measures (*p* < 0.01, Cohen’s d = 2.23), muscular endurance (*p* < 0.05; Cohen’s d = 1.74), and physical fitness (*p* < 0.05, Cohen’s d = 1.39) The TUG and TUDS scores increased respectively by 15% and 12%, muscular endurance by 24%, and physical fitness by 5%
Post et al. (2022)^31^ United States of America	Quasi- experimental	**IG:** 6 men and 5 women clinically diagnosed with Down syndrome Age: 25.8 ± 6.4 years IQ: 58.3 ± 19.7 units	10-week resistance training exercise protocol	**Mode:** Light Repetition Maximum (RM) (12–15 RM), moderate (8–10 RM), and heavy (4–6 RM) for three sets. It included: warm-ups from large to smaller muscle groups (leg press, bench press, leg curl, shoulder press, bicep curls, and variations to make it enjoyable each day). Exercises were performed with machines, free weights, resistance bands, and body weight alone **Duration:** 45–60 min/session for 10 weeks	**Motor Function Testing:** The TGMD-2 (short sprint, gallop, shuffle, stationary basketball dribble, catch a baseball) **Flexibility:** sit-and-reach test **Muscular Strength:** 6-repetition maximum testing for the leg press, and bench press, as well as the 30-s chair sit-to-stand test **Muscular endurance:** 30-s of push-ups and 30-s of sit-ups	Participants significantly improved both locomotor skill (*p* = 0.001), object control skill (*p* = 0.008), and total gross motor function (p = 0.000) This intervention significantly improved sit-and-reach flexibility, 6 RM barbell bench press, 6 RM supine leg press, 30-s push-ups, 30-s sit-ups, and 30-s chair sit-to-stand metrics
Cai and Baek (2022)^29^ China	Experimental	22 adults (18 men and 4 women) with DS between 18 to 40 years **IG:** 11 participants **CG:** 11 participants	**IG:** Basketball programme, strictly supervised, conducted in a 28-m long, 15-m wide, international standard basketball court of Basketball **CG:** Performed no structured exercises	**Mode:** Basketball programme - Warm up with games for 10 min - 45 min of basic basketball skill learning (shooting, passing and handling) and physical training - Relaxing exercises for 5 min **Frequency:** 3 times/week **Duration:** 60 min/session for 24 consecutive weeks	**Aerobic fitness:** 16-m modified shuttle-run test or PACER test **Balance:** - Static: standing on one leg to a maximum period of 10 s - Dynamic: number of consecutive steps (max six steps) walking on a 3.05-m balance beam that was 10.2 cm wide **Muscular strength:** - As many curls up as possible (maximum of 75) sliding their hands up along the upper legs to the kneecap - Standing long jump (explosive force of the lower limbs): Distance between the initial line to the heel after jumping **Flexibility:** The sit-and-reach test	After 24 weeks there was a statistically significant improvement in all functional health variables in the IG (all featured *P* < 0.05) The CG had no statistically significant difference between all functional health variables except for the standing long jump (all featured *P* > 0.05) The IG had better flexibility, sit and-reach, modified curl-up, aerobic capacity and static and dynamic balance than the control group (*P* < 0.05)

*IG* Intervention group, *CG* Control group, *DS* Down Syndrome, *IQ* Intelligence quotient, *EMG* Electromiography, *ACSM* American College of Sports Medicine, *GMFM-88* The Gross Motor Function Measure 88, *6MWD* 6-min walking distance test, *ADL’S* Activities of daily living. *TUG*: The Timed Up and Go test, *TUDS* Timed Up and Down Stairs test, *TGMD-2* Test of Gross Motor Development version 2.

### Risk-of-bias assessment

**Figure 2:**
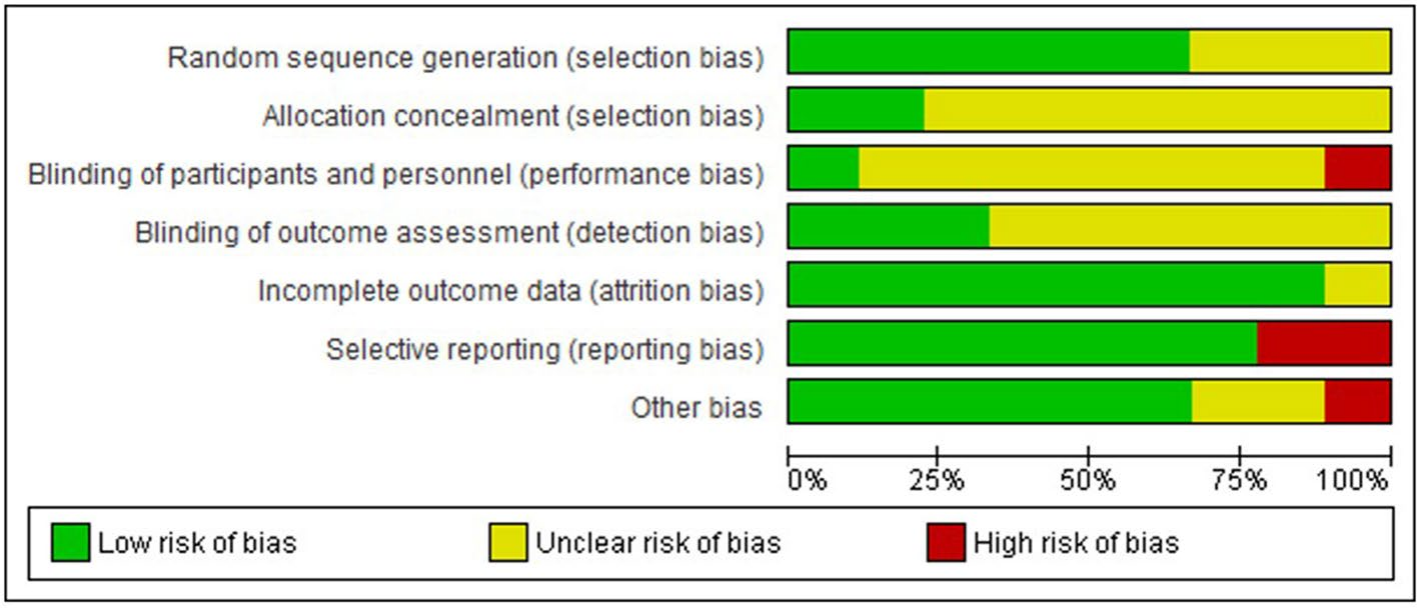
Risk of bias graph.

**Figure 3:**
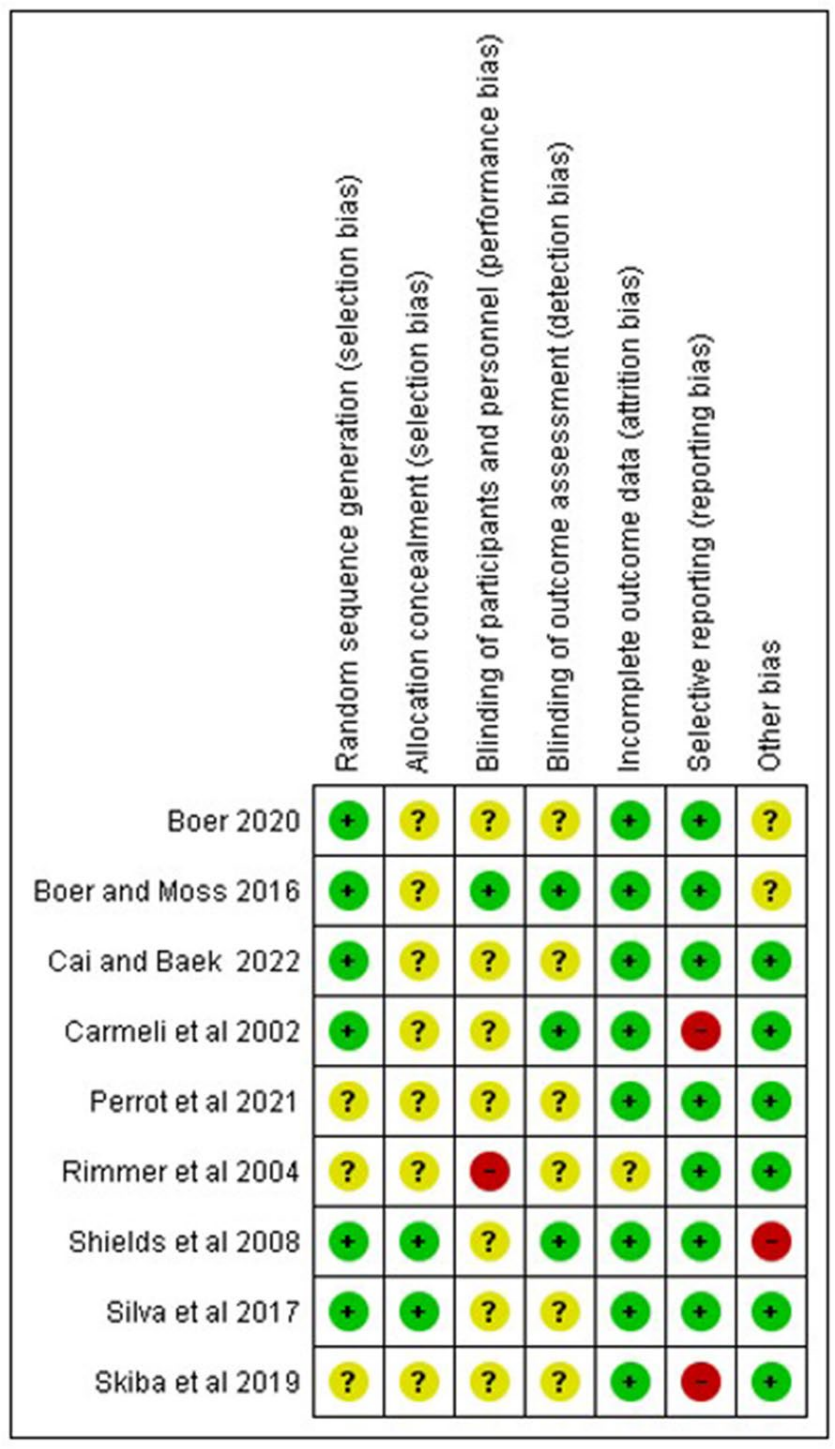
Risk of bias summary.

All included studies had some risk of bias. Three studies presented an unclear risk because there was randomization, but the method was not described^17,20,32^. Related to blinding only one study^20^ had high risk bias because participants were notified of the group they belonged to with no mention of personnel blinding. Six studies^1,17,20,32,33,36^ had unclear bias in blinding of outcome assess. Just one study^20^ had unclear risk of bias of incomplete outcome data and only two studies^14,17^ obtained a high risk of bias in selective outcome reporting. In other aspects assess, one study^12^ presented a high risk of bias due to possible confounding bias. Figures 2 and 3 summarize the information on bias risk domains.

The methodological quality of the seven selected quasi-experimental studies^2,13,19,31,34,35,37^ was moderate to serious Table 2.

**Table 2:** ROBINS-I (risk of bias judgements in non-randomized studies of interventions).

	Confounding	Selection of participants	Classification of interventions	Deviations from intended interventions	Missing data	Measurement of outcomes	Selection of reported results	Overall
Davis y Sinning 1987^33^	Moderate	Low	Moderate	Low	Low	Serious	Low	Serious
Aguiar et al. 2008^27^	Low	Low	Low	Low	Moderate	Moderate	Moderate	Moderate
Cowley et al. 2011^9^	Moderate	Low	Low	Low	Moderate	Serious	Low	Serious
Mendonca et al. 2011^15^	Low	Low	Low	Low	Moderate	Moderate	Low	Moderate
Boer y de Beer 2019^2^	Low	Low	Low	Low	Low	Moderate	Low	Moderate
Tsimaras V et al. 2004^30^	Low	Low	Low	Low	Low	Moderate	Low	Moderate
Post E et al. 2022^31^	Moderate	Low	Low	low	low	Serious	Low	Serious

Low comparable to a well-performed randomized trial; Moderate sound for a non-randomized study, but not comparable to a rigorous randomized trial; Serious presence of important problems; Critical too problematic to provide any useful evidence on the effects of intervention; Overall risk of bias equal to the most severe level of bias found in any domain.

### Therapeutic exercise interventions used for each outcome

#### Muscular strength

This outcome was the most frequently reported within the studies included. The interventions addressed aerobic exercise in water^1,2^, continuous and interval aerobic exercise^5^, aerobic exercise^14,32,33^, progressive resistance exercise^12,13^, resistance exercise^35,37^, and combined exercise^19,20,33,34^. The duration of the interventions was between 6^2^ and 25 weeks^14^ with frequencies ranging from two^12,13,32^ to three times a week^1,2,5,14,19,20,33,34,37^. The measuring instruments are shown in Table 1.

Results were found in favor of interventions for abdominal strength^1^, increasing the number of abdominal push-ups performed. However, these results were not significant (SMD = 0.39[95% CI  − 0.38–1.17]). A trend was found in favor of the experimental groups in the lower limb test (Appendix 1), where the number of times to get up and sit down on a chair in 30 s was increased^1,5^. However, there were no significant results after these interventions (SMD = 0.15 [95% CI −0.37–0.68]).

Finally, significant results were found in the strength of both the upper (SMD = 0.74 [95% CI 0.25–1.22]) and lower limbs (SMD = 0.56 [95% CI 0.08–1.04]), regarding the interventions of combined exercise^20^ and progressive resistance exercise^12^ against the muscular strength of the upper and lower limbs (Appendix 1), with increases in the 1-repetition maximum (RM) leg press and 12-RM chest press protocols.

Quasi-experimental studies using resistance^13,35,37^, or aerobic exercise^1,2,34^ also showed statistically significant differences in increasing muscle strength (Table 1).

### Balance

Interventions used to improve balance included aerobic exercise in aquatic environment^1,2^ and in the terrestrial environment^14,32,33^, continuous and interval aerobic exercise^5^, and combined exercise^34,36^. These interventions lasted between 6^2^ and 25 weeks^14^, with a frequency of two^32^ to three times per week. The measuring instruments are shown in Table 1.

Both static and dynamic balances were assessed. For static balance, Boer et al.^1^ observed improvements in time maintaining balance on one leg and in the number of consecutive steps on a balance beam; however, these improvements were not significant. Regarding dynamic balance, the improvements were not significant either^36^. Carmeli et al.^14^ with aerobic exercise reported significant improvements in dynamic balance between groups *p* < 0.05 and finally, Cai and Baek^33^ with a combined exercise plan showed significant increase of dynamic and static balance.

A quasi-experimental studies using aerobic exercise^34^ also showed statistically significant differences in dynamic balance (Table 1).

### Gait

Two studies evaluated the effectiveness of their gait interventions^13,17^. The interventions applied were aerobic exercise through Nordic walking^17^ and progressive resistance exercise^13^. The measuring instruments are shown in Table 1. The interventions lasted 10 weeks with a frequency of two^13^ to three times per week^16^. Cowley et al.^13^ found no significant differences in gait speed after their intervention. Conversely, Skiba et al.^16^ found an improvement in the space–time parameters, angular changes of the limbs, and mean values of the angular deviations at the joints.

### Functional tasks

The interventions applied included aerobic exercise in an aquatic^1,2^ and terrestrial^32^ environment, continuous and interval aerobic exercise^5^, progressive resistance exercise^12,13^, and combined exercise^36^. The durations ranged from 6^2^ to 14 weeks^32^, with frequencies ranging from two times^12,13,32^ to three times per week^1,2,5,36^. The measuring instruments are shown in Table 1.

It was found that the interventions applied for this outcome^1,5^ had not significant results (SMD = 0.47 [95% CI =  − 0.07,1.01]) in the 6-min walk performance (Appendix 1). Similarly, the time to perform the test after the interventions was reduced in the 8-foot up and go test^1,5^. However, these results were not significant (SMD =  − 0.48 [95% CI  − 1.04–0.07]) (Appendix 1).

Perrot et al.^32^ in a randomized clinical trial showed significant improvements in functional task using combined exercise via Nintendo Wii (Table 1).

Just one quasi-experimental study using resistance exercise^13^ showed significant improvements in go up and down 10 steps test, or aerobic exercise also showed statistically significant differences in increasing muscle strength (Table 1).

### Assessment of the certainty of the evidence

It was found that the certainty of evidence was low for all studies because some had a high risk of bias in the random sequence generation and an unclear risk in blinding. In other studies, the confidence intervals were wide and exceeded the no effect line and some had unclear risk of bias in 4 out of 7 criteria (Table 3).

**Table 3:** Assessment of the certainty of evidence presented for each outcome.

Evaluation of certainty							Summary of outcomes				
No. of studies	Study design	Risk of bias	Inconsistency	Indirect evidence	Imprecision	Other considerations	No. of patients		Effect		Certainty
							Therapeutic exercise	Control	Relative (95% CI)	Absolute (95% CI)	
Aerobic exercise. Muscular strength – Sit-to-stand test (monitoring range 6–12 weeks; assessed with Number of repetitions)											
2	RCT^1,5^	not serious^a^	not serious	not serious	serious^b^	none	26	29	–	SMD 0.15 higher (− 0.48;1.27)	⨁⨁⨁◯ Moderate
Combined exercise. Muscular strength—1 RM Leg press (monitoring range 10–12 weeks; assessed with Weight Lifted)											
2	RCT^8,16^	Serious^c^	not serious	not serious	serious^b^	none	39	33	–	SMD 0.56 higher (0.08;1.04)	⨁⨁◯◯ Low
Combined exercise. Muscular strength—1 RM chest press (monitoring range 10–12 weeks; assessed with Weight lifted)											
2	RCT^8,16^	Serious^c^	not serious	not serious	serious^b^	none	39	33	–	SMD 0.73 higher (0.25;1.22)	⨁⨁◯◯ Low
Balance											
4	RCT^1,10,29,32^	Serious^a^	not serious	not serious	serious^b^	none	54	47	Carmeli et al^10^ with aerobic exercise reported significant improvements in dynamic balance between groups p < 0.05. Cai et al.^29^ with a combined exercise plan showed significant increase of dynamic and static balance		⨁⨁◯◯ Low
Aerobic exercise. Gait											
1	RCT^13^	Serious^a^	not serious	not serious	not serious	none	11	11	Skiba et al. found an improvement in the space–time parameters, angular changes of the limbs, and mean values of the angular deviations at the joints		⨁⨁⨁◯ Moderate
Combined exercise. Functional Fitness—6 min walking distance (6MWD) (monitoring 14 weeks)											
1	RCT^28^	Serious^a^	not serious	not serious	Not serious^a^	none	6	6	Perrot et al. showed significant improvements in functional task using combined exercise via nintendo wii		⨁⨁⨁◯ Moderate

*RCT* Randomized clinical trial.

^a^Unclear risk of bias in masking.

^b^Wide confidence intervals that pass the line of no effect.

^c^Unclear risk of bias in 4 of 7 criteria.

## Discussion

This systematic review assessed the effect of different types of physical exercise on the motor function of adult DS individuals. Among the types of exercise identified in the literature reviewed to improve muscle strength, balance, and gait are aerobic exercise in different modes, such as aquatic, judo, bicycle, and walking. Modes with mechanotherapy equipment were included within the muscle strength exercise, and endless treadmills and elliptical bike were mainly included within the cardiovascular resistance exercises.

Physical exercise is important throughout life because it improves the health^38^. Physical exercise also has a therapeutic objective for DS individuals as they may require exercise-based clinical interventions throughout their life to improve physical abilities, such as muscle strength, flexibility, and balance^39^. These diminished physical abilities in DS individuals hinder the performance of ADL and impact their quality of life^40^.

Three studies included muscle strength as an outcome. Aguiar et al.^31^ found no differences between the initial and final measurements after a judo training program, while Boer^1^ and Boer and de Beer^2^ observed an increase in muscle strength with aquatic aerobic exercise, as did Boer and Moss^5^ with a bike or treadmill workout. However, the impact of aerobic exercise on increasing muscle strength remains controversial^41^. The American College of Sports Medicine recommends combining the intensity, volume, and frequency of training to improve muscle strength in young, middle-aged, and older populations and to optimize muscle hypertrophy and strength gains^38,42^. Vigorous training intensity and/or high training frequency, however, may be difficult to include in a training program for DS individuals owing to their characteristic comorbidities as well as in older adults.

Studies in other populations have reported improvements in muscle hypertrophy, and thus, in muscle strength among younger and older adults after engaging in a single type and mode of physical exercise that includes walking^43,44^ and riding a bicycle^45,46^, which is consistent with the findings of this review.

Aerobic exercise and the exercise combination were used to improve balance in adults with DS in aquatic mode^2^, with treadmill^14^, basketball^33^ and bicycle^5^, and differences were reported only for the intervention in aquatic mode and with exercise combination. Earlier studies have shown the mechanisms underlying this type of exercise that can improve balance, considering that the aquatic environment can stimulate an increase in the strategies and postural adjustments necessary for the execution of different movements^47^. This improves the quality of motor function by improving muscle strength and balance^48^.

The ability to walk and do ADL depends on being able to control dynamic and static balance, thus requiring the control of the trajectory of center of pressure^49^. Improvements in balance after resistance exercise have been attributed mostly by enhanced muscle strength, neural function and force control, as muscle torque is required to maintain balance^50^, thus, exercise training without a specific balance component may be effective in improving balance control due to a direct influence on muscle mass and strength. It would be important to consider the specificity principle of training, improvements in balance would be amplified by adding balance training in both static and dynamic conditions using neuromuscular exercise in combination with aerobic or resistance exercise (neuromuscular exercise)^51^.

There were no differences in functional fitness between aerobic exercise in the aquatic or terrestrial modes. Studies including resistance exercise did not assess this outcome. Functional fitness is a construct in which all the physical abilities, which were also included as outcomes in this review, including muscle strength, balance, and posture, and other outcomes, which were not prioritized in this review, such as flexibility and mobility, were included. Physical exercise should enhance all of these abilities for an impact on functional fitness^49^. On the other hand, the benefits of strength may not transfer effectively to concomitant improvements in functional outcomes such as balance, functional tasks, or activities of daily living^52^. The contradictory evidence may be due to differences in training intensity, frequency, type of resistance training, equipment (such as free weights or resistance machines), and types of measurements used to evaluate balance, strength and ADL.

Among the limitations of this study, we can mention the methodological limitations of the studies included. All the studies included met at least one of the criteria for unclear risk of bias and 9 out of 12 studies met at least one criterion for high risk of bias. This is consistent with the certainty of evidence for each of the outcomes proposed in this study, as it was low for all the outcomes included. This is partly attributable to the methodological limitations already stated, but also to the imprecision of the studies, possibly due to the small sample sizes included in the primary studies^53^.

On the other hand. The meta-analyses report very low statistical heterogeneity or in some cases 0 according to the I^2^, although there is clearly clinical heterogeneity in the included primary studies. These meta-analyses were performed with a small number of primary studies, which may explain the results obtained in the assessment of heterogeneity. It should be taken into account that within the statistical test used to assess heterogeneity is χ^2^ (Chi-square) and the inconsistency index or I^2^. Both tests are influenced by the number of studies included in the meta-analyses, therefore, it is possible that the limited number of studies influence the results of the test and these are interpreted as homogeneous when in fact the opposite may occur. It is necessary that the reader and decision maker keep this information in mind when analyzing the findings of systematic reviews with meta-analyses^54^.

The strengths of this systematic review are having analyzed the effectiveness and prescription parameters of the different types of therapeutic exercise that can be used in people with DS. As a limitation, no sensitivity analysis was performed nor was publication bias explored due to the small sample size of the studies and the low number of studies included in the meta-analysis.

Future research can study the effect of different types of exercise on clinical rehabilitation goals among adult DS individuals. Studies with robust research designs and sample sizes consistent with the effect measure are required to evaluate the effects of exercise on the physical abilities of DS individuals.

## Conclusions

There is low certainty of the evidence for the outcomes proposed in this review. Therapeutic exercise, however, could be effective in improving muscle strength and gait functionality. Standardized instruments that measure the outcomes in motor function and research of better methodological quality that assess the effectiveness of the exercise prescription parameters are required. This would facilitate the evaluation of the effectiveness of the intervention as well as decision-making in the practice regarding the type of exercise that would be indicated for each patient according to his or her therapeutic needs.

### Supplementary Information

Supplementary Information.

## Data Availability

The datasets used and/or analyzed during the current study available from the corresponding author on reasonable request.

## Abbreviations

DS: Down syndrome
ADL: Activities of daily living
RM: Repetition maximum
RoB: Risk-of-bias
MD: Mean difference
