# Supplementary material for: Effects of therapeutic exercise on the motor function of adults with Down syndrome: a systematic review and meta-analysis
Source: Sci Rep. 2023 Dec 11;13:21962. doi: 10.1038/s41598-023-48179-1 (PMC10713621; doi:10.1038/s41598-023-48179-1)
Supplement: Supplementary file 1 — Supplementary Information. [file 41598_2023_48179_MOESM1_ESM.docx]

Appendix 1.


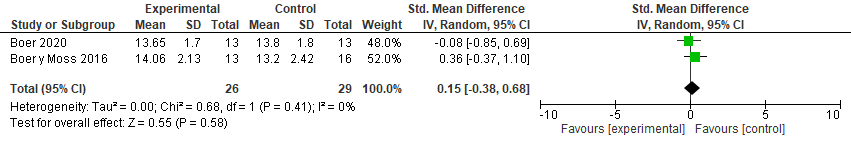


**Figure 4.** Aerobic exercise in water versus aerobic exercise on a firm surface, outcome: Muscular strength – Sit to Stand Test


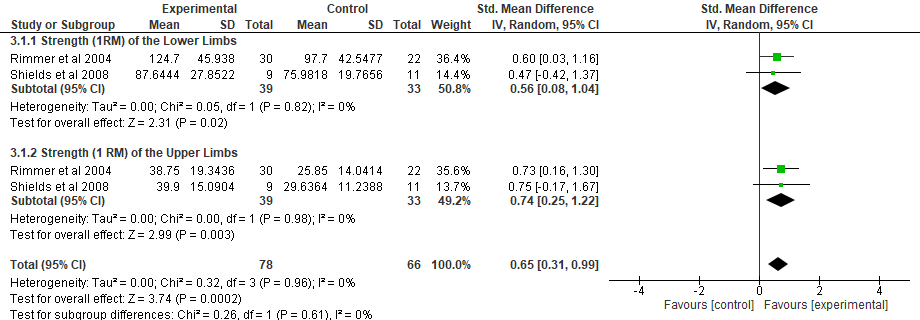


**Figure 5.** Combined exercise versus progressive resistance exercise, outcome: Strength of the Lower and Upper Limbs – 1RM


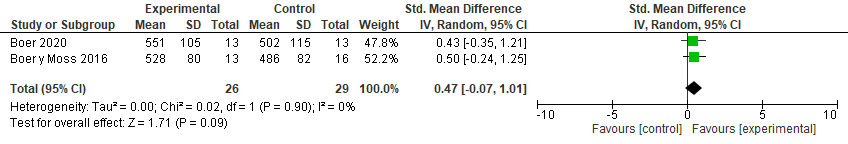


**Figure 6.** Aerobic exercise in water versus aerobic exercise on a firm surface, outcome: Functional Tasks - 6MWD.


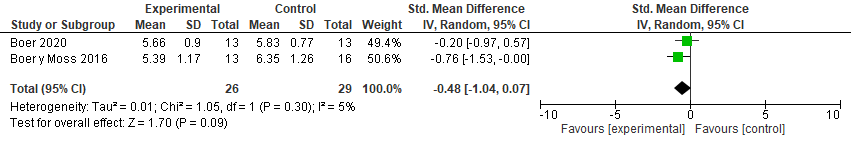


**Figure 7.** Aerobic exercise in water versus aerobic exercise on a firm surface, outcome: Functional Tasks - 8 ft up and go.
